# Crystal structures of N-terminal WRKY transcription factors and DNA complexes

**DOI:** 10.1007/s13238-019-00670-0

**Published:** 2019-11-16

**Authors:** Yong-ping Xu, Hua Xu, Bo Wang, Xiao-Dong Su

**Affiliations:** State Key Laboratory of Protein and Plant Gene Research, and Biomedical Pioneering Innovation Center (BIOPIC), Peking University, Beijing, 100871 China

**Dear Editor,**

Plant-specific WRKY transcription factors (TFs) are among the largest families of TFs in higher plants; they are also found in the unicellular eukaryote *Giardia lamblia* and the slime mold *Dictyostelium discoideum* (Ulker and Somssich, 2004), but not in animals. WRKY TFs participate in diverse developmental and physiological processes in plants, such as disease resistance, abiotic stress responses, senescence, seed and trichome development, as well as additional developmental and hormone-controlled processes (Agarwal et al., 2011).

There are 75 WRKY family members identified so far in *Arabidopsis* and more than 100 in rice (UniProt:http://www.uniprot.org/). The WRKY TFs are named after their approximately 60 conserved amino acids of DNA binding domains (DBDs) called the WRKY domains required for W-box (5′-TTGAC-C/T-3′) DNA recognition (Eulgem et al., 2000). The WRKY domain contains a highly conserved WRKYGQK motif (forming a β strand) near the N-terminus and a zinc-finger motif at the C-terminus, featuring an atypical C2H2 (CX_4–5_CX_22–23_HX_1_H) or C2HC (CX_7_CX_23_HXC) type (Eulgem et al., 2000). The zinc-finger structure is indispensable for the DNA-binding of WRKY TFs. Any substitutions of the conserved cysteine or histidine residue eliminate the protein-DNA interaction (Duan et al., 2007). Based on the number of WRKY domains and the zinc-finger structure, WRKY TFs are classified into groups I to III and each group is further divided into subgroups (Brand et al., 2013). Group I WRKY genes are defined by the presence of two WRKY domains, whereas groups II and III contain only a single WRKY domain (Eulgem et al., 2000). We named the WRKY domains as WRKY-N and WRKY-C respectively for the group I two-domain WRKY TFs. As shown by previous experiments, the specific binding to W-box was thought to be mediated mainly by the C-terminal WRKY domain, whereas the N-terminal WRKY domain showed weaker (Brand et al., 2013) or even no binding to W-box (Ishiguro and Nakamura, 1994; de Pater et al., 1996; Eulgem et al., 1999). However, recent one-hybrid studies on yeast demonstrated that the two WRKY domains of *At*WRKY1 (*Arabidopsis* WRKY1 protein) were both essential for its transcriptional activities (Qiao et al., 2016). We have previously determined the crystal structure of the apo *At*WRKY1-C (*Arabidopsis* WRKY1-C) that comprises a five-stranded β-sheet (Duan et al., 2007). The solution structures of the apo *At*WRKY4-C (*Arabidopsis* WRKY4-C), and its complex with a W-box DNA were solved by NMR (Yamasaki et al., 2005; Yamasaki et al., 2012). Recently, a complex crystal structure of dimeric rice WRKY45-DBD, a group III (C2HC zinc finger domain) WRKY TF bound to two W-box DNA was reported (Cheng et al., 2019). To date, there is no structural information for any N-terminal WRKY domains.

In this study, we present the crystal structures of *At*WRKY1-N (residues 108–169) at 3.0 Å, *At*WRKY2-N (residues 270-332) at 2.4 Å and *At*WRKY33-N (residues 185–242) at 3.0 Å in complex with a same 15-bp W-box double stranded DNA (dsDNA) (Tables S1 and S2). The three complex structures look very similar to each other and all comprise four β strands named β2 to β5, forming a β sheet (partially consistent with the other reported WRKY structures), and the sheet inserted almost vertically into the major groove of the W-box DNA (Fig. 1A–D). The notable changes of these three DNA complex structures are the orientation of the loop linking β4-β5 compared with other structures of *At*WRKY1-C (PDB code: 2AYD), *At*WRKY4-C (PDB code: 2LEX), *Os*WRKY45 (PDB code: 6IR8) and *At*WRKY52 (PDB code: 5W3X) (Fig. 1E). The most striking differences are that the distances (measured between main chain CO and NH groups) between β3 and β4 of these three *At*WRKY-N proteins are closer than those of other WRKY domains (mean distance 3.0 Å for *At*WRKY-N proteins vs. 5–6 Å for the others); Fig. 1F and 1G showed two sets of comparisons between group I WRKY proteins. As mentioned above, the *Os*WRKY45-DBD can form a homodimer by swapping the β4-β5 strands (Cheng et al., 2019). From above comparison, we could suggest that the domain swapping of *Os*WRKY45-DBD is relevant to the flexibility around β3 and β4 strands.

**Figure 1:**
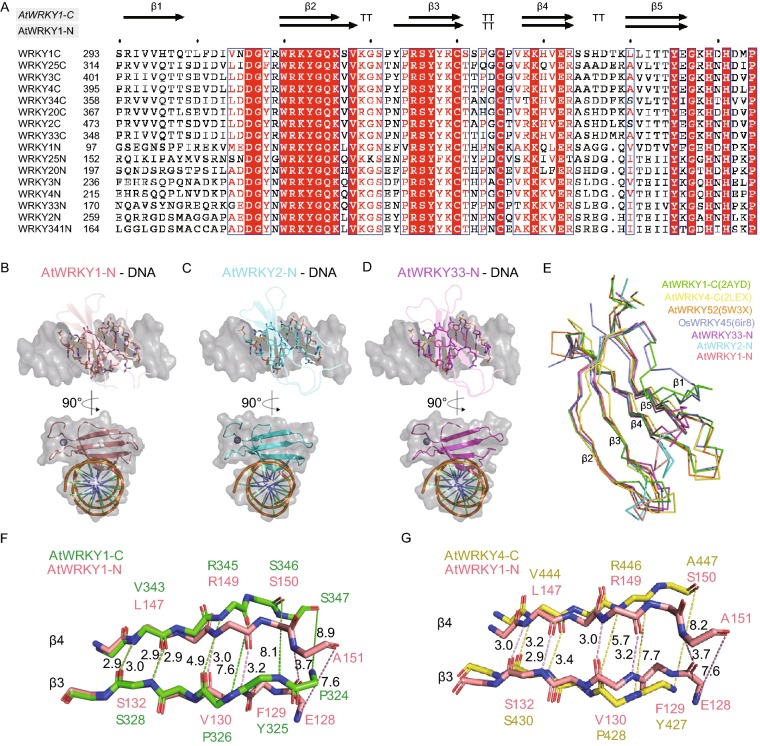
**Overall properties and structures of N-terminal WRKY TFs**. (A) Sequence alignments of DBDs of both the N-terminus and C-terminus of eight *Arabidopsis* WRKY proteins from group I based on the crystal structure of AtWRKY1-C (PDB code: 2AYD) (Duan et al., 2007) and AtWRKY1-N complexed with W-box DNA. Completely conserved residues are highlighted with red boxes, whereas less conserved residues are painted with different colors. (B–D) Overall structures of the N-terminal WRKY proteins (AtWRKY1-N, AtWRKY2-N and AtWRKY33-N) in complex with W-box DNA are shown in two orientations. The direct contacting residues with DNA are shown as sticks. (E) Structural comparison of three N-terminal WRKY proteins and other four WRKY domain structures. The loops between β4 and β5 strands with different conformations are circled by the gray background. (F and G) Distances of β3 and β4 of AtWRKY1-N are compared with those of AtWRKY1-C and AtWRKY4-C respectively. Their distances (Å) are shown next to

The residues directly interacting with the dsDNA are conserved in all three complex structures (Fig. 2A), and they interact with DNA in the same manner, either specific base recognition (Fig. S1) or nonspecific interaction to the main chain phosphate groups. We will take the structure of *At*WRKY1-N to describe the details below. Besides the previous reported recognition of the W-box sequence by *At*WRKY4-C mainly via apolar contacts with the methyl groups of the TT bases (Yamasaki et al., 2012) (Fig. 2C and 2D), our crystal structures showed extensive H-bond interactions between *At*WRKY1-N and dsDNA with recognition of the Crick strand G6’, G7’ and C9’ (using prime “ base’ ” to denote the 5′ -> 3′ reading from the Crick strand**)** (Fig. 2B and 2E). In our structure, most of distances between amino acids (*At*WRKY1-N) and bases are closer (>0.2 Å) than those of *At*WRKY4-C (Fig. 2B–E). Accordingly, the *At*WRKY1-N contributes to DNA binding with higher affinity of *K*_D_ ~0.1 µmol/L (Fig. 2G), whereas the *K*_D_ for the *At*WRKY1-C is 1.3 µmol/L (Fig. S2B) measured by ITC (isothermal titration calorimetry) assay, a 13-fold increase in the DNA binding affinity.

**Figure 2:**
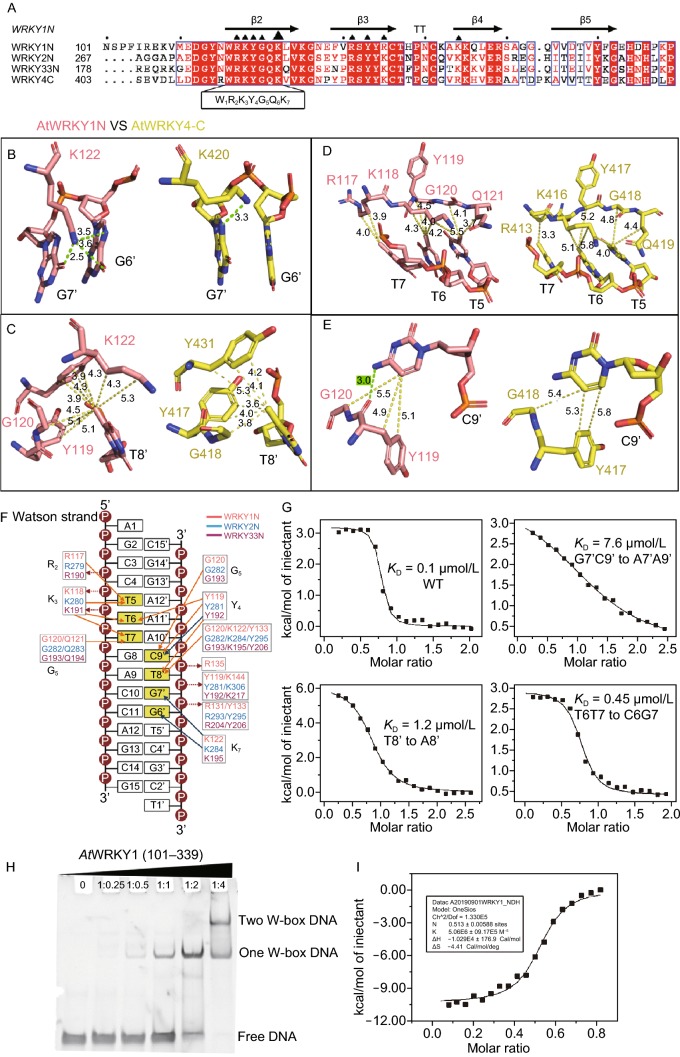
**Detailed protein-DNA interactions of AtWRKY-1N**. (A) Structure-based sequence alignments of AtWRKY1-N, AtWRKY2-N, AtWRKY33-N and AtWRKY4-C based on AtWRKY1-N structure. The residues for recognizing DNA are marked by black triangles, and the sizes of the triangles correspond to the importance of the interacting residues. The highly conserved WRKYGQK sequence is coded as W_1_R_2_K_3_Y_4_G_5_Q_6_K_7_. **(**B–E) The comparison of interacting details of amino acids and DNA bases between AtWRKY1-N and AtWRKY4-C. Their distances (Å) are shown next to. The green dashed lines indicate H-bonds and the yellow is hydrophobic interactions. (F) Schematic representation of the interactions of AtWRKY1-N, AtWRKY2-N and AtWRKY33-N with the consensus W-box DNA sequences. Interactions of amino acid residues with phosphate groups and nucleobases are shown as red dotted lines and solid lines, respectively. H-bonds are indicated by blue, and the apolar contacts are orange. (G) ITC experiments of AtWRKY1-N and the dsDNA containing the W-box motif and the mutated sequences. The full dsDNA sequences used in the ITC experiments were shown in Table S2, and the affinity comparation was summarized in Table S3. (H) The EMSA results of AtWRKY1^101−339^ (residues 101–339, comprising both WRKY domains) binding to W-box DNA. Molar ratios of protein-to-DNA are shown at the top of each gel as the molar concentration of protein increases gradually. The DNA sequence is shown in Table S2. The first band indicates both WRKY domains bind to DNA at the same time whereas the second band denotes only one WRKY domain participates in DNA binding. (I) ITC experiments were performed by titrating 0.11 mmol/L AtWRKY1^101−339^ into 0.026 mmol/L W-box DNA. The number of N equals 0.5 indicating the two WRKY domains of AtWRKY1^101−339^ (residues 101–339) can interact with DNA at the same time. The DNA sequence used in the ITC experiment is shown in Table S2

The DNA recognition was accomplished by seven base-specific interactions (Watson strand T5, T6, T7 and Crick strand G6’, G7’, T8’, C9’) and nonspecific interactions with the phosphate backbone (Fig. 2F). To provide a unified description for all WRKY domains with different numbering, we renumbered the highly conserved WRKYGQK sequence as W_1_R_2_K_3_Y_4_G_5_Q_6_K_7._ The base-recognition of Watson strand are mainly by hydrophobic interactions, including: (a) R_2_ and K_3_ to T5, (b) K_3_ and Y_4_ to T6, (c) K_3_, G_5_ and Q_6_ to T7 (Fig. 2F). In addition, the side chain of R_2_ and K_3_ contact the phosphate backbone of C4, T5 and T6 through nonspecific salt bridges and hydrogen bonds (Fig. 2F). As for the Crick strand, there are more interactions involved in DNA recognition. The hydrophobic interactions include: (a) G_5_, Y_4_, K_7_ and Y133 to T8’, (b) Y_4_ and G_5_ to C9’. There also exist H-bonds, including: (a) the amino-group of K_7_ forms hydrogen bonds with the O6 and N7 atoms of G6’ and G7’, (b) the carboxyl oxygen of Y_4_ and the N4 atom of C9’ (Fig. 2F). Meanwhile, the H-bonds and electrostatic interactions between protein and DNA phosphate groups strengthen the binding preference to the sequence -G6’G7’T8’C9’- of the Crick strand. The H-bonds include: (a) the guanidyl of R131 and the hydroxyl of Y133 to the G7’, (b) the hydroxyl of Y_4_ and the side chain of K144 to T8’, (c) the guanidyl of R135 to C9’. Electrostatic interactions are found between Arg or Lys and DNA phosphate group, including R131 to G7’, K144 to T8’ and R135 to C9’ (Fig. 2F).

We also studied the contacts described above by base substitution and evaluated their binding affinities by ITC measurements (Fig. 2G). The binding affinity of the substitutions of G7’C9’ to A7’A9’ is much weaker than in the original sequence. The *K*_D_ between *At*WRKY1-N and G7’C9’ to A7’A9’ is 7.6 µmol/L, whereas the *K*_D_ between *At*WRKY1-N and the original W-box is 0.1 µmol/L, a 76-fold decrease (Fig. 2G and Table S3), showing the importance of these two bases for the specific recognition. In addition, the substitution of T8’ to A8’ significantly decreased the affinity by 12-fold (Fig. 2G and Table S3). However, the substitution of G6’ to T6’ only decreased the binding affinity by 2.5-fold (Fig. S2A and Table S3). Subsequently, we mutated the T6 and T7 of the Watson strand to C6 and G7, which only decreased the affinity by 4.5-fold (Fig. 2G and Table S3). Therefore, TT bases of the Watson strand are not as important to *At*WRKY1-N as to *At*WRKY4-C (Yamasaki et al., 2012) and OsWRKY45-DBD (Cheng et al., 2019). These results demonstrate that the DNA-protein interaction of AtWRKY1N is mainly concentrated on the Crick strand particularly around sequence of “**-**G7’T8’C9’-”. The substitution of the bases outside the “**-**G7’T8’C9’-” only impairs the binding slightly (Table S3). A previous work also revealed that the DBDs of *At*WRKY11 and *At*WRKY50 bind to an invariant ‘GAC’ core consensus (reading from the Watson strand) (Brand et al., 2013), consistent with our results.

Next, we investigated the residues of *At*WRKY1-N involved in DNA binding by site-directed mutagenesis and electrophoretic mobility shift assay (EMSA). The mutant of R117A (R_2_) or K118A (K_3_), interacting with TT bases of the Watson strand, could still bind to DNA (Fig. S3A and S3B), whereas the mutation of K416A (K_3_) in *At*WRKY4-C eliminated its DNA binding activity (Yamasaki et al., 2012). The mutants Q121A (Q_6_), K122A (K_7_), Y133A, R135A and K144A appeared to not bind to DNA without an apparent shift band (Fig. S3D, S3E and S3G–I), noticeably the mutant K122A (K_7_), with Y119 (Y_4_), Q121 (Q_6_), K122 (K_7_), Y133, R135 and K144 directly in contact with the sequence G7’, T8’, C9’ and T7 (Fig. 2F). The mutant Y119A still bound to DNA (Fig. S3C) because Y119 (Y_4_) forms a hydrogen bond with base C9’ via the main chain oxygen atom (Fig. 2E). These results are consistent with the complex structures observed above and ITC results.

The classical model of a transcription factor searching for its specific site presumes that positively charged DBD binds first to dsDNA somewhere non-specifically and then slides on the DNA in one dimension to find the specific site (Berg et al., 1981). In our case, the residues involved in non-specific contacts surrounding the phosphate groups appear to enable the protein to locate closer to the DNA major groove non-specifically, and the K_7_ contributes to searching for the optimal specific binding site. However, we could not obtain the dynamic process from the static picture of our crystal structures. The residue K_7_ is absolutely conserved for all WRKY proteins. We thus propose that K_7_ is the key amino acid for all WRKY domains to search for and bind to dsDNA specifically. In our three complex structures, the K_7_ interacts with G6’ and G7’ with different but similar distances (Fig. S4A–C). To understand the role of K_7_ in different WRKY domains, we mutated it to Ala, Gln and Arg. Only the mutant K284R of AtWRKY2-N could form a slight band with DNA in one of the WRKY domains while the other mutants completely eliminated the DNA binding ability (Fig. S4D–F).

All together, we have shown that the N-terminal group I WRKY domains bind to W-box DNA as well (if not better) as the C-terminal WRKY domains, with quite different binding mode (more extensive interaction to the Crick strand and to the ‘GAC’ core sequence). Furthermore, the EMSA and ITC results show that *At*WRKY1^101−339^ (residues 101–339, comprising both WRKY domains) can bind to two W-box DNA at the same time (Fig. 2H–I). The *K*_D_ between *At*WRKY1^101−339^ and W-box DNA is 0.5 µmol/L with two DNA binding sites (Fig. 2I). The mechanism of two binding-sites on group I WRKY proteins immediately suggests that group I WRKY TFs can interact and recruit more DNA partners than previous knowledge of a single domain of WRKY TF binding to one W-box DNA (Fig. S5).

WRKY TFs bind to DNA specific sites in the promoters of target genes to regulate their expression. However, all WRKY TFs bind to the same W-box sequence, raising the question of how specificity is achieved and differentiated between different promoters and WRKY TFs. The differences in their binding site preferences were suggested to partly depend on flanking sequences outside the TTGACY-core motif (Ciolkowski et al., 2008). Our study also emphasized that N-terminal WRKY domain interacting with W-box is more concentrated on a conserved ‘-G’T’C’-’ consensus on the Crick strand (Figs. 2F, 2G and S2A), indicating some diversity in the binding sequences since there should be many more binding sites with the three bases ‘-G’T’C’-’ (or ‘GAC’ reading from the Watson strand) consensus. A WRKY gene from *Tamarix hispida*, *Th*WRKY4, could bind to two other motifs: a W-box like sequence (GTCTA) and the RAV1A element (CAACA) (Xu et al., 2017). The former consists of the invariant ‘GTC’ motif while another is a novel sequence. These studies suggest and in agreement with our results that the WRKY TFs not only recognize the conventional W-box (TTGACC), but also could bind to other DNA sequences.

## Electronic supplementary material

Below is the link to the electronic supplementary material.

Supplementary material 1 (PDF 839 kb)
