# Supplementary material for: Crystal structures of N-terminal WRKY transcription factors and DNA complexes
Source: Protein Cell. 2019 Nov 16;11(3):208–13. doi: 10.1007/s13238-019-00670-0 (PMC7026264; doi:10.1007/s13238-019-00670-0)
Supplement: Supplementary file 1 — Supplementary material 1 (PDF 839 kb) [file 13238_2019_670_MOESM1_ESM.pdf]

Supplementary Information

Crystal Structures of N-terminal WRKY transcription factors and DNA complexes

Yong-ping Xu [1], Hua Xu [1], Bo Wang[1] and Xiao-Dong Su[1*]

[1] State Key Laboratory of Protein and Plant Gene Research, and Biomedical Pioneering Innovation Center (BIOPIC), Peking University, Beijing, 100871, China.

[*] Lead contact

Correspondence and requests for materials should be addressed to X.D.S. (email: xdsu@pku.edu.cn).

Keywords: N-terminal WRKY domain, DNA-protein interaction, X-ray crystallography, binding affinity

Running title: Crystal structures of N-terminal WRKY domains and DNA complexes

Contents

MATERIALS AND METHODS

Supplementary Table 1-3

Supplementary Figure1-5

MATERIALS AND METHODS

Sequence alignments. CLUSTAL OMEGA (Sievers et al., 2011) was used for multiple sequence alignments among the N-terminal and C-terminal WRKY domain using default parameters, and the results were displayed by ESPript 3.0201 (Robert and Gouet, 2014). The aligned sequences included the Arabidopsis N-terminal and C-terminal WRKY domain of WRKY1 (At2g04880; UniProt ID: Q9SI37), WRKY2 (At5g56270, UniProt ID:Q9FG77), WRKY3 (At2g03340, UniProt ID:Q9ZQ70), WRKY4 (At1g13960, UniProt ID:Q9XI90), WRKY20 (At4g26640, UniProt ID:Q93WV0), WRKY25 (At2g30250, UniProt ID: O22921), WRKY33 (At2g38470, Q8S8P5), and WRKY34 (At4g26440, O65590).

Protein expression and purification. All proteins were overexpressed and purified with the same protocol. The codon-optimized DNA binding domains (DBDs) of the Arabidopsis WRKY domain were constructed into the pET21b vector with C-terminal his-tag and subsequently transformed into the E. coli strain BL21 (DE3). Protein overexpression was induced by the addition of isopropyl β-D-1-thiogalacto-pyranoside to a final concentration of 0.5 mM. Cells were left to grow overnight at 18 ℃. Bacteria were then collected and resuspended in buffer A (25 mM HEPES, pH 7.0, 1.0 M NaCl) before sonication and centrifugation. Subsequently, the supernatant was loaded onto a Ni-chelating column (GE Healthcare, USA), and target proteins were eluted by imidazole at a concentration of 200–500 mM, followed by size-exclusive chromatography (Superdex 75, GE Healthcare) for final purification in buffer C (25 mM HEPES, pH 7.0, 100 mM NaCl). Purified proteins were concentrated and stored at -80 ℃ after flash freezing by liquid nitrogen.

Crystallization and structure determination. W-box DNA (Supplementary Table 2) for crystallization was purchased from ThermoFisher (Thermo Fisher Scientific, USA) and dissolved in buffer C (25 mM HEPES, pH 7.0, 100 mM NaCl). All DNA samples were heated to 95 ℃, slowly cooled down to anneal to room temperature, and then further purified by size-exclusive chromatography (Superdex 75, GE Healthcare) to remove single-stranded DNA. Then, 10 mg/mL WRKY1N, WRKY2N or WRKY33N was mixed with 10 mg/mL W-box DNA. After incubating on ice for 20 minutes, the complexes were mixed with an equal volume of reservoir at 18 ℃ using the sitting drop method. The complex crystals of WRKY1N-DNA, WRKY2N-DNA, and WRKY33N-DNA were grown in Crystal Screen 2-13 (0.2 M ammonium sulfate, 0.1 M sodium acetate trihydrate (pH 4.6)，30% polyethylene glycol monomethyl ether 2000, Hampton Research), ProPlex^{TM} 1-28 (0.2 M lithium sulfate, 0.1 M MES (pH 6.0), 20% PEG 4000, Molecular Dimensions) and Wizard Classic 3-12 (8% v/v ethylene glycol, 0.1 M HEPES/sodium hydroxide (pH 7.5), 10% PEG 8000, Rigaku). The crystals were then soaked in crystallization solutions with 20% glycerol for several minutes before being flash frozen in liquid nitrogen. All diffraction data were collected at beamline BL19U at the National Facility for Protein Science Shanghai using a PILATUS 6M detector. The datasets were processed with the XDS (Kabsch, 2010) program package, and the structure was determined by molecular replacement in Phaser-MR with the model 2ayd (PDB ID) and a B-form DNA (automatic generated by Coot (Emsley and Cowtan, 2004)) using the PHENIX program suite (Adams et al., 2010). The structure model was improved by Coot (Emsley and Cowtan, 2004) and refined by phenix.refine (Adams et al., 2010). The crystallographic parameters of the model are listed in Supplementary Table 1. All structure figures were generated by PyMOL2.0 (Delano Scientific).

Electrophoretic mobility shift assays (EMSA). The W-box DNA were annealed to form a Cy3-labeled DNA duplex. The typical binding reaction (6 ul) contained dsDNA (6 pmol), 25 mM HEPES (pH 7.0), 100 mM NaCl, 5% glycerol and gradient-purified proteins while the control group had no protein. The binding reaction mixture was incubated at 4 ℃ for 20 minutes, and the complex was loaded on

a 10% nondenaturing polyacrylamide gel electrophoresis (PAGE) in 0.5×TBE at 120 V for 1 hour. Lastly, the fluorescence was detected using AI600 (GE Healthcare, USA).

Isothermal Titration Calorimetry (ITC) Assays. The DNA preparation followed the same protocol as in the crystallization session. The proteins and DNA were in buffer C (25 mM HEPES, pH 7.0, 100 mM NaCl) before titration. To determine the affinities between proteins and DNA, 0.25 mM protein was titrated into 0.026 mM DNA using an ITC200 (GE Healthcare) at 20 ℃. The thermograms were integrated by Origin software and fitted in the "one set of sites" mode.

Accession numbers

The accession numbers of the PDB codes for the structures of the WRKY1-N, WRKY2-N and WRKY33-N in complex with the W-box DNA reported in this paper are 6J4E, 6J4F and 6J4G.

Reference

Adams, P.D., Afonine, P.V., Bunkoczi, G., Chen, V.B., Davis, I.W., Echols, N., Headd, J.J., Hung, L.W., Kapral, G.J., Grosse-Kunstleve, R.W., *et al.* (2010). PHENIX: a comprehensive Python-based system for macromolecular structure solution. Acta Crystallogr D Biol Crystallogr *66*, 213-221.

Emsley, P., and Cowtan, K. (2004). Coot: model-building tools for molecular graphics. Acta Crystallogr D Biol Crystallogr *60*, 2126-2132.

Kabsch, W. (2010). Xds. Acta Crystallogr D Biol Crystallogr *66*, 125-132.

Robert, X., and Gouet, P. (2014). Deciphering key features in protein structures with the new ENDscript server. Nucleic Acids Res *42*, W320-324.

Sievers, F., Wilm, A., Dineen, D., Gibson, T.J., Karplus, K., Li, W.Z., Lopez, R., McWilliam, H., Remmert, M., Soding, J., *et al.* (2011). Fast, scalable generation of high-quality protein multiple sequence alignments using Clustal Omega. Mol Syst Biol *7*.

Supplementary Table 1 | Data collection and refinement statistics.

	WRKY1N-DNA	WRKY2N-DNA	WRKY33N-DNA
Data Collection Statistics			
Space Group	$P6_522$	P1	$P6_522$
Wavelength (Å)	0.9778	0.9778	0.9778
Number of Reflections	134,594(13,523)	61,112 (6,492)	138,619(13,228)
Cell dimensions			
a, b, c (Å)	99.0, 99.0, 114.6	38.1, 48.3, 69.1	97.9, 97.9, 94.3
α, β, γ (°)	90, 90, 120	93.7, 87.3, 97.5	90, 90, 120
Resolution (Å)	28.58 - 3.10	29.58 - 2.40	29.47 - 3.00
	(3.21 - 3.10)	(2.49 - 2.40)	(3.11 - 3.00)
Rmerge (%)	15.3 (84.4)	5.8 (38.9)	16.3 (80.0)

I/σ (I)	18.4 (3.81)	9.6 (2.1)	17.5 (1.5)
Completeness (%)	98.4 (96.1)	93.48 (96.37)	98.83 (100.00)
Multiplicity	20.9 (22.2)	3.4(3.6)	24.4 (24.1)
Wilson B-factor	79.27	52.60	82.13
Refinement Statistics			
Reflections used in refinement	6349 (586)	17785 (1804)	5670 (550)
Reflections used for Rfree	294 (24)	1790 (174)	567 (55)
Rwork/Rfree %	0.20/0.25 (0.38/0.42)	0.19/0.23 (0.30/0.37)	0.21/0.25 (0.34/0.46)
Number of non-hydrogen atoms	1118	2267	1089
Macromolecules	1117	2257	1088
Zn+	1	2	1
Solvent	-	8	-
Protein residues	62	126	59
B factor			
Protein	75.51	65.63	72.45
Ligands	66.04	48.38	71.27
Solvent	-	59.79	-
R.m.s deviations			
Bond Lengths (Å)	0.012	0.011	0.010
Bond Angles (°)	1.39	1.13	1.17
Ranachandran (%)			
Preferred region	93.33	95.08	92.86
Allowed region	6.67	4.92	7.14
Outliers	0.00	0.00	0.00

Values in bracket indicate those of the highest resolution shell.

Supplementary Table 2 | DNA sequences used in each assay.

DNA	Sequence
DNA for crystallization	5’ AGCCTTTGACCAGCG 3’<br>3’ CGGAAACTGGTCGCT 5’
W-box DNA for ITC	5’ CGCCTTTGACCAGCGC 3’<br>3’ GCGGAAACTGGTCGCG 5’
W-box DNA for EMSA	5’cy3- CGCCTTTGACCAGCGC 3’<br>3’ GCGGAAACTGGTCGCG 5’
T8’ to A8’	5’ CGCCTTTGTCCAGCGC 3’<br>3’ GCGGAAACAGGTCGCG 5’

G7'C9' to A7'A9'	5' CGCCTTTTATCAGCGC 3'
	3' GCGGAAAATAGTCGCG 5'
G6' to T6'	5' GCGCTTGTCAAAGGCG 3'
	3' CGCGAACAGTTTCCGC 5'
T6T7 to C6G7	5' CGCCTCGGACCAGCGC 3'
	3' GCGGAGCCTGGTCGCG 5'

Supplementary Table 3 | DNA binding affinity compared with some substitutions.

Position of substitution	K_D (μM)	Decrease (-fold)
W-box DNA for ITC	0.1	-
G7'C9' to A7'A9'	7.6	76
T8' to A8'	1.2	12
T6T7 to C6G7	0.45	4.5
G6' to T6'	0.25	2.5

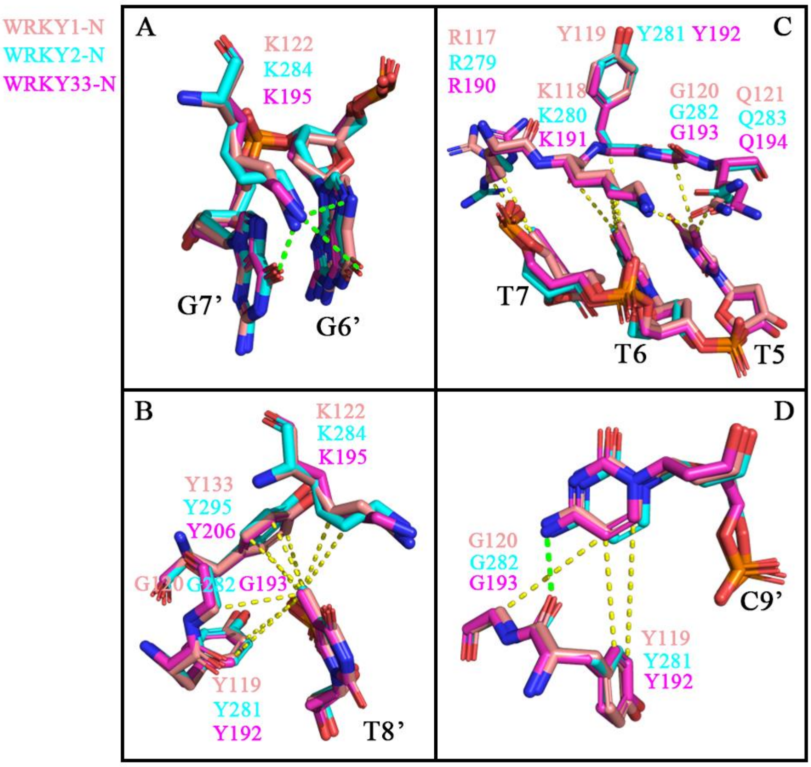

Supplementary Figure 1 | The comparations of amino acids involved in bases recognition of the three complex structures. The amino acids participating in specific DNA interactions are aligned together by Pymol. The DNA bases and the main chain of the amino acids overlap very well. And the side chains of amino acids have a little deviation (< 0.2 Å). The cartoon of the WRKY1-N, WRKY2-N, and WRKY33-N are colored in pink, cyan, and magenta respectively. The numbers of amino acids are shown next. The green dashed lines indicate H-bonds and the yellow is hydrophobic interactions. The distances can refer to Fig. 2B-2E.

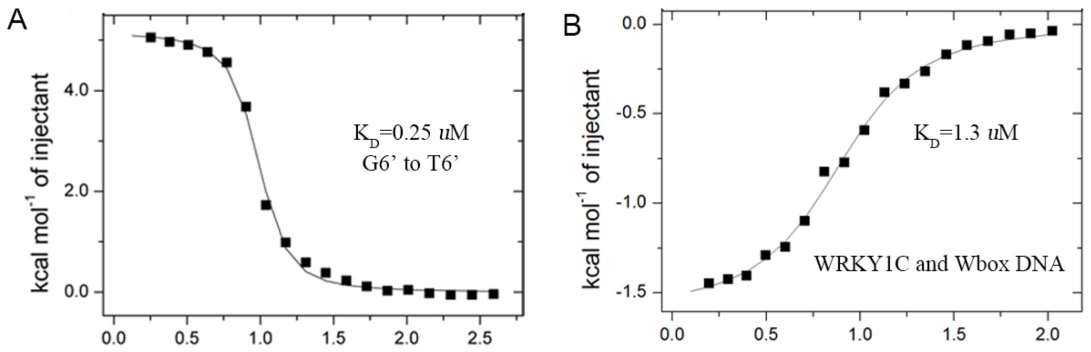

Supplementary Figure 2 | ITC experiments of AtWRKY1-N and AtWRKY1-C with the dsDNA containing the mutated sequence and W-box motif respectively. A, ITC experiments were performed by titrating AtWRKY1-N into the dsDNA with mutation. The full dsDNA sequences used in the ITC experiments were shown in Supplementary Table 2, and the affinity comparation was summarized in Supplementary Table 3. B, ITC experiments were performed by titrating AtWRKY1-C into the W-box DNA.

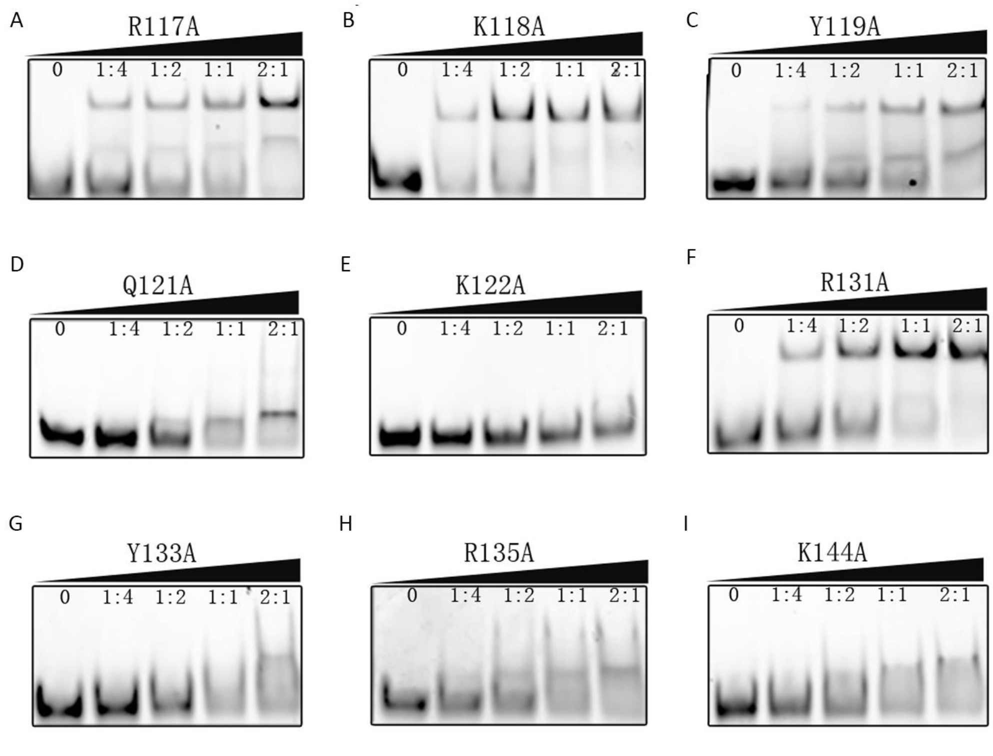

Supplementary Figure 3 | EMSA experiments of the mutants of AtWRKY1-N involved in dsDNA recognition with the W-box DNA. The dsDNA sequences were shown in Supplementary Table2. Molar ratios of protein-to-DNA are shown at the top of each gel as the molar concentration of protein increases gradually. The R117A means the residue Arg117 was mutated to Ala. The other mutants were named similarly.

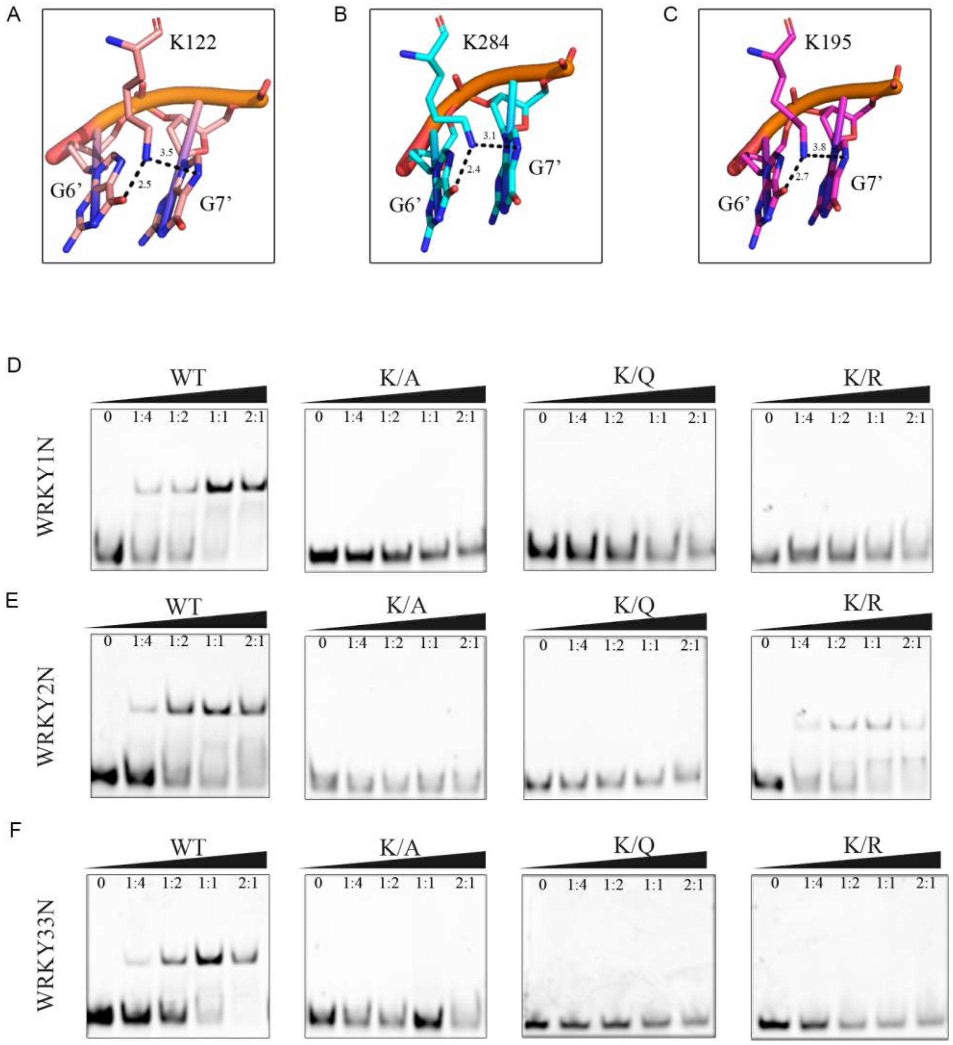

Supplementary Figure 4 | The key amino acid of K_7 for specific binding. A-C, The nucleobases recognition of G6’ and G7’ by the K_7 of $W_1R_2K_3Y_4G_5Q_6K_7$. A-C represent WRKY1-N, WRKY2-N, WRKY33-N in turn. D-F, EMSA experiments of the mutants of K_7 with the W-box DNA. The residue of K_7 was mutated to Ala, Gln, and Arg. The DNA sequences are shown at Supplementary Table 2. Molar ratios of protein-to-DNA are shown at the top of each gel as the molar concentration of protein increases gradually.

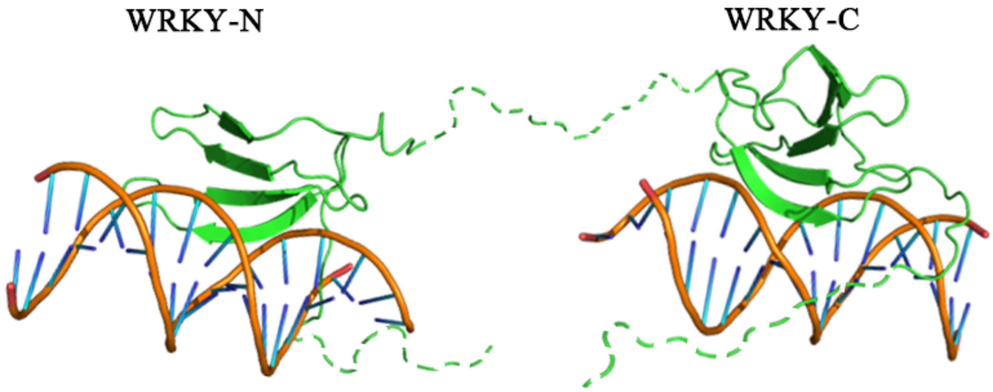

Supplementary Figure 5 | Proposed interaction model for Group I double DBD WRKY TFs and W-box DNA. The model suggests that group I WRKY TFs can recruit and bring together more DNA-binding sites with two WRKY domains.
